# FLAIR and ADC Image-Based Radiomics Features as Predictive Biomarkers of Unfavorable Outcome in Patients With Acute Ischemic Stroke

**DOI:** 10.3389/fnins.2021.730879

**Published:** 2021-09-16

**Authors:** Guanmin Quan, Ranran Ban, Jia-Liang Ren, Yawu Liu, Weiwei Wang, Shipeng Dai, Tao Yuan

**Affiliations:** ^1^Department of Medical Imaging, The Second Hospital of Hebei Medical University, Shijiazhuang, China; ^2^GE Healthcare China, Beijing, China; ^3^Department of Clinical Radiology, Kuopio University Hospital, Kuopio, Finland; ^4^Department of Radiology, Handan Central Hospital, Handan, China; ^5^Department of Radiology, Cangzhou City Hospital, Cangzhou, China

**Keywords:** acute ischemic stroke, outcome, magnetic resonance imaging, apparent diffusion coefficient, radiomics

## Abstract

At present, it is still challenging to predict the clinical outcome of acute ischemic stroke (AIS). In this retrospective study, we explored whether radiomics features extracted from fluid-attenuated inversion recovery (FLAIR) and apparent diffusion coefficient (ADC) images can predict clinical outcome of patients with AIS. Patients with AIS were divided into a training (*n* = 110) and an external validation (*n* = 80) sets. A total of 753 radiomics features were extracted from each FLAIR and ADC image of the 190 patients. Interquartile range (IQR), Wilcoxon rank sum test, and least absolute shrinkage and selection operator (LASSO) were used to reduce the feature dimension. The six strongest radiomics features were related to an unfavorable outcome of AIS. A logistic regression analysis was employed for selection of potential predominating clinical and conventional magnetic resonance imaging (MRI) factors. Subsequently, we developed several models based on clinical and conventional MRI factors and radiomics features to predict the outcome of AIS patients. For predicting unfavorable outcome [modified Rankin scale (mRS) > 2] in the training set, the area under the receiver operating characteristic curve (AUC) of ADC radiomics model was 0.772, FLAIR radiomics model 0.731, ADC and FLAIR radiomics model 0.815, clinical model 0.791, and clinical and conventional MRI model 0.782. In the external validation set, the AUCs for the prediction with ADC radiomics model was 0.792, FLAIR radiomics model 0.707, ADC and FLAIR radiomics model 0.825, clinical model 0.763, and clinical and conventional MRI model 0.751. When adding radiomics features to the combined model, the AUCs for predicting unfavorable outcome in the training and external validation sets were 0.926 and 0.864, respectively. Our results indicate that the radiomics features extracted from FLAIR and ADC can be instrumental biomarkers to predict unfavorable clinical outcome of AIS and would additionally improve predictive performance when adding to combined model.

## Introduction

Acute ischemic stroke (AIS) is a critical cerebrovascular disorder worldwide with high morbidity and disability and accounts for 60–80% of all strokes (Darwish et al., 2020). The cerebral infarct in the middle cerebral artery (MCA) territory represents the most frequent AIS (Sommer et al., 2018). Early diagnosis and prognosis assessment are crucial in the management of AIS. At present, the National Institute of Health Stroke Scale (NIHSS) is the most commonly used clinical score for the evaluation of functional impairment (Choi et al., 2015). Most of the clinical trials on AIS are based on computed tomography (CT), CT angiography (CTA), and CT perfusion (CTP), which provide several and fast information about cerebral ischemic tissue. However, contrast-enhanced CT techniques are not universally accepted methods in the routine workup in AIS patients in some institutions due to the possible risk of intravenous injection of iodinated contrast agent and technical complex. Instead, non-contrast CT and multimodality magnetic resonance imaging (MRI) are used as substitute imaging modalities for clinical evaluation in AIS patients. Conventional MR stroke protocol, even without contrast injection, could be an alternative tool for providing both anatomic and functional information, including the lesion location and size, occluded vessels, diffusion characteristics, and cerebral blood perfusion obtained by arterial spin label (ASL) technique. Thus, the objective MRI markers would be useful to assist in predicting prognosis for an individual AIS patient.

By intravenous injection of contrast agent, enhanced MRI can provide further information, and the lesion mismatch on perfusion-weighted imaging (PWI) and diffusion-weighted imaging (DWI) has been employed to estimate the ischemic penumbra (Darwish et al., 2020). Whereas, enhanced MRI is not a universally accepted method in the routine workup in AIS patients due to an intravenous injection of gadolinium agent, it is time consuming, and the patient’s cooperation. Evidences have also shown its possibility to improve prognostic estimation with other non-enhanced MRI features, including fluid-attenuated inversion recovery (FLAIR) vascular hyperintensity (FVH) (Jiang et al., 2019), susceptibility vessel sign (SVS) (Rudilosso et al., 2021), cerebral artery laterality (Ichijo et al., 2013), and lesion volume (Bucker et al., 2017). Nevertheless, most of these studies are based only on morphologic information. Therefore, we will focus on FLAIR and apparent diffusion coefficient (ADC) as our research sequences in the setup of prognosis prediction models in this study.

As a novel developed data analysis technique, radiomics can extract great many quantitative features from medical images. Radiomics features would reflect subtle pathophysiologic features and heterogeneity of lesions. Radiomics has been employed in the diagnosis, prognostic estimation, and treatment evaluation of varied diseases. A recent study presented a promising result by using texture analysis with FLAIR and DWI images in AIS patients (Wang et al., 2020). They found that the texture features could be used as biomarkers to assess stroke severity. In other studies, radiomics features were extracted from CT angiography, enhanced T1WI, and ADC map. It has been proven that radiomics is an effective image analysis tool in the depiction of ischemic penumbra (Tang et al., 2020), evaluation of collateral circulation (Dolotova et al., 2020), prediction of hemorrhagic transformation (Kassner et al., 2009), prediction of malignant infarct (Wen et al., 2020), and forecasting clinical outcome in AIS patients. However, prognosis estimation was often made only based on radiomics features without clinical factors. So far, few studies have been performed to setup a prognostic model based on radiomics to predict clinical outcome individually. We hypothesized that the radiomics features extracted from FLAIR and ADC could be a prognostic biomarker for predicting clinical outcome in AIS patients.

Thus, the aim of this study was to explore radiomics based on FLAIR and ADC to predict the clinical outcome after 90 days of AIS onset. In addition, we tested the diagnostic performance of this model in an external validation set.

## Materials and Methods

### Patients

This study was approved by the ethics committee of The Second Hospital of Hebei Medical University. Due to its retrospective nature, informed consent from patients was waived. AIS patients were retrospectively collected from three hospitals during January 2018 to June 2020. AIS was defined as the presence of acute clinical vascular syndromes and infarction DWI in MCA territory. Inclusion criteria included the following: (1) a first-ever AIS in unilateral MCA territory; (2) acute ictus of stroke ≤ 72 h before MR examination; (3) the maximum diameter of AIS lesions ≥ 1 cm; and (4) modified Rankin scale (mRS) at 90 days after ictus was available. Exclusion criteria included the following: (1) lacunar cerebral infarct; (2) secondary hemorrhagic transformation of AIS lesions; (3) coexisting other diseases that affect neurologic function, such as brain tumor, cerebral hemorrhage, trauma, and demyelinating disease; (4) age less than 18 years old; and (5) severe artifact on FLAIR or ADC images.

**FIGURE 1:**
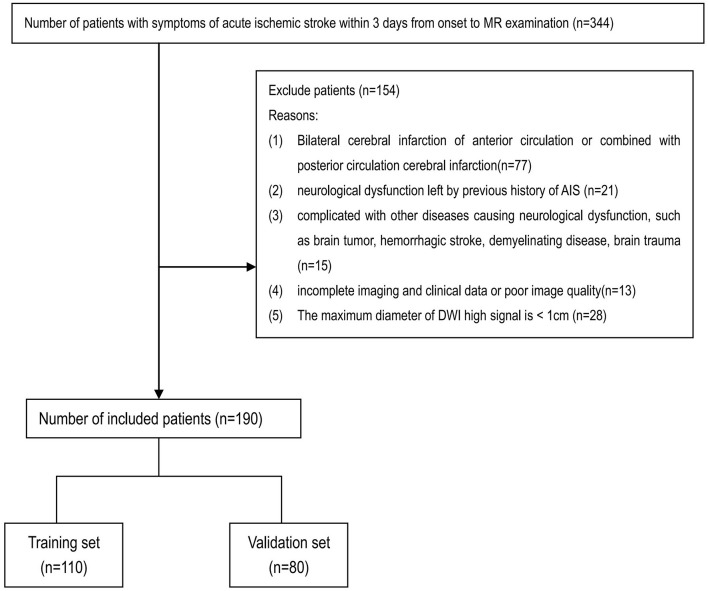
Flowchart of the collection of patients.

During the study period, 344 AIS patients were collected. There were 154 patients excluded due to bilateral cerebral infarction of anterior circulation or combined with posterior circulation cerebral infarction (*n* = 77); neurological dysfunction left by previous history of AIS (*n* = 21); complicated with other diseases causing neurological dysfunction, including brain tumor, hemorrhagic stroke, demyelinating disease, and brain trauma (*n* = 15); incomplete imaging and clinical data or poor image quality (*n* = 13); and the maximum diameter of DWI high signal < 1 cm (*n* = 28). Finally, 190 patients were enrolled into the present study (Figure 1). According to the Transparent Reporting of a multivariable prediction model for Individual Prognosis or Diagnosis (TRIPOD) (Collins et al., 2015), 110 patients who came from one hospital were assigned as training set. Eighty patients who came from the other two hospitals were assigned as external validation set. There was no significant difference in the baseline clinical and MRI variables between two sets (Table 1). A comparison of population characteristics of anterior circulation AIS patients with different outcomes is shown in Table 2.

**TABLE 1:** Population characteristics of patients with AIS in the training and validation sets.

**Characteristics**	**Training (*n* = 110)**	**Validation (*n* = 80)**	***p*-value**
Age (years), median (IQR)	62 (50.75, 70.25)	58 (50, 68)	0.586
Male sex, *n* (%)	78 (70.9%)	61 (76.3%)	0.412
**Risk factors, *n* (%)**			
Hypertension	69 (62.7%)	52 (65.0%)	0.748
Diabetes mellitus	28 (25.5%)	15 (18.8%)	0.276
Hyperlipidemia	6 (5.5%)	6 (7.5%)	0.567
Coronary heart disease	9 (8.2%)	7 (8.8%)	0.889
Smoking	35 (31.8%)	28 (35.0%)	0.646
Alcohol consumption	29 (26.4%)	14 (17.5%)	0.149
Previous stroke attack, *n* (%)	16 (14.5%)	11 (13.8%)	0.877
Interval from ictus to MR (hours)	26.50 ± 15.77	28.69 ± 14.36	0.627
Orthogonal diameter of the lesion, median (IQR)	2.47 (1.14, 6.66)	3.82 (1.51, 8.73)	0.107
Admission NIHSS, median (IQR)	3 (2, 6.25)	4 (2, 8)	0.077
Fazekas scores, median (IQR)	2.5 (1, 4)	2 (1, 3)	0.305
Admission DWI-ASPECTS, median (IQR)	9 (8, 9)	8 (7, 9)	0.105
mRS at 90 days, median (IQR)	2 (1,3)	1 (1,3)	0.200
mRS > 2, *n* (%)	30 (27.27%)	21 (26.25%)	0.875

*DWI-ASPECTS, diffusion-weighted imaging-Alberta Stroke Program Early CT Score; mRS, modified Rankin score; *n*, number; NIHSS, National Institute of Health Stroke Scale.*

**TABLE 2:** Population characteristics of AIS patients with favorable and unfavorable outcome.

**Characteristics**	**Favorable (*n* = 139)**	**Unfavorable (*n* = 51)**	***p*-value**
Age (years), median (IQR)	56 (48, 65.5)	69(59, 75)	<0.001*
Male sex, *n* (%)	110 (79.1%)	29 (56.9%)	0.002*
**Risk factors, *n* (%)**			
Hypertension	91 (65.5%)	30 (58.8%)	0.399
Diabetes mellitus	31 (22.3%)	12 (23.5%)	0.858
Hyperlipidemia	11 (7.9%)	1 (2%)	0.153
Coronary heart disease	9 (6.5%)	7 (13.7%)	0.111
Smoking	45 (32.4%)	18 (35.3%)	0.705
Alcohol consumption	33 (23.7%)	10 (19.6%)	0.546
Previous stroke ictus, *n* (%)	17 (12.2%)	10 (19.6%)	0.197
Interval from ictus to MR (hours)	26.85 ± 13.65	29.26 ± 15.75	0.268
Orthogonal diameter of the lesion, median (IQR)	2.32 (1.12, 5.28)	7.99 (15.05, 2.67)	<0.001*
Admission NIHSS, median (IQR)	3 (2, 5)	7 (5, 10.5)	<0.001*
Fazekas scores, median (IQR)	2 (1, 3)	2 (1, 4)	0.369
Admission DWI-ASPECTS, median (IQR)	9 (8, 9)	8 (6, 9)	<0.001*
mRS at 90 days, median (IQR)	1 (0, 2)	3 (3, 4)	<0.001*

DWI-ASPECTS, diffusion-weighted imaging-Alberta Stroke Program Early CT Score; mRS, modified Rankin score; *n*, number; NIHSS, National Institute of Health Stroke Scale. *, with signifcant difference.

The demographic and clinical data (Liu et al., 2018) included gender, age, history of hypertension, diabetes mellitus, hyperlipidemia, coronary heart disease, smoking, alcohol consumption, previous cerebral infarct attack, time from ictus to MR examination (in hours), baseline NIHSS score on admission, and mRS score at 90 days. The orthogonal diameters (ODs) of infarct lesion (Yuan et al., 2019), admission addressStreetDiffusion-Weighted Imaging-Alberta Stroke Program Early CT (DWI-ASPECT) score, and Fazekas score (Fazekas et al., 2002) were measured for each patient. Unfavorable clinical outcome was defined as mRS > 2 at 90 days (Jiang et al., 2019). The treatment strategy included intravenous thrombolytic therapy, anti-thrombotic therapy, anticoagulant, oral statins, and antiplatelet. Arterial thrombectomy was performed in 12 patients in this cohort.

### Image Analysis

Acute ischemic stroke patients underwent MR examination with a 3T Philips MR scanner (Achieva, Netherlands; in The Second Hospital of Hebei Medical University) or a GE MR System (Signa, United States; in Handan Central Hospital and Cangzhou City Hospital). The MRI protocol included FLAIR, DWI, and magnetic resonance angiography (MRA). The imaging protocol parameters at the Philips MR system were as follows: (1) FLAIR: repetition time (TR)/echo time (TE) = 9,000 ms/140 ms, inversion time (TI) = 2,600 ms; (2) DWI: single-shot echo planar imaging, *b* = 1,000 s/mm^2^, TR/TE = 2,208 ms/96 ms; and (3) MRA: time of flight sequence, 3D acquisition, TR/TE = 20 ms/3.5 ms. Imaging parameters at the GE MR system were as follows: (1) FLAIR: TR/TE = 8,000 ms/126 ms, TI = 2,400 ms; (2) DWI: single-shot echo planar imaging, *b* = 1,000 s/mm^2^, TR/TE = 4,500 ms/72.5 ms; and (3) MRA: time of flight sequence, 3D acquisition, TR/TE = 20 ms/3.2 ms.

The conventional MRI factors, including ODs of the lesion, Fazekas scores, and admission DWI-ASPECTS, were analyzed by two neuroradiologists (with 10 and 21 years of experience separately) independently. When a disagreement existed, consensus was reached after consulting another radiologist with a 26-year experience in neuroradiology. The ODs were measured on the DWI slice with the largest lesion diameter (Yuan et al., 2019). If there were multiple infarct lesions, the ODs of the first three largest lesions were summed up. We recorded the DWI-ASPECTS, in which a score of 0 indicates diffuse infarct throughout the MCA territory and a score of 10 represents no lesion (Tei et al., 2011). The white matter lesions were evaluated with Fazekas score (Fazekas et al., 2002), in which a score of 3 indicates confluent lesions and a score of 0 represents no lesions.

### Feature Extraction

**FIGURE 2:**
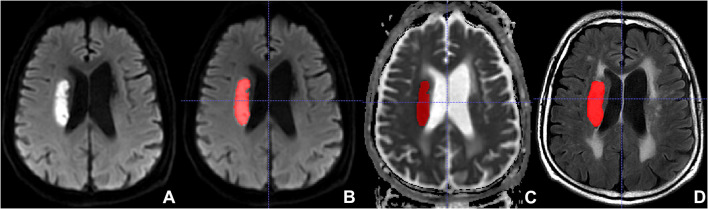
Lesion segmentation with the Insight Segmentation and Registration Toolkit-ANAP (ITK-SNAP). **(A)** Non-segmented diffusion-weighted imaging (DWI). **(B)** Segmented DWI. **(C)** Segmented apparent diffusion coefficient (ADC). **(D)** Segmented fluid-attenuated inversion recovery (FLAIR).

The FLAIR and ADC images of all eligible AIS patients were imported into the software Insight Segmentation and Registration Toolkit-ANAP (ITK-SNAP, version 3.8.0^1^). Two neuroradiologists manually delineated the lesions on the ADC, then the region of interest (ROI) on ADC was copied to the corresponding FLAIR slice by using a free available software^2^ (Figure 2). In the areas of infarction, the FLAIR and ADC images were consequently segmented and were loaded into the open-source platform, PyRadiomics^3^, to extract radiomics features (van Griethuysen et al., 2017). Logistic regression analysis was employed for further selection of significant features. These selected significant features were used to calculate radscore.

### Development and Validation of Prediction Models

To develop prediction models, we performed a univariate logistic regression analysis for each potential factor (age, gender, various risk factors of cerebral vascular disease, interval from ictus to MR, ODs of infarct, admission NIHSS, Fazekas score, and admission DWI-ASPECTS) in the training set to select demographic, clinical, and conventional MRI predictors associated with unfavorable outcome. Then, the factors showing significant in the univariate logistic regression analysis and the radiomics features were used to develop six prognosis prediction models by using multivariable logistic regression analysis: ADC radiomics model, FLAIR radiomics model, ADC + FLAIR radiomics model, clinical model, clinical + conventional MRI factors model, and a combination model (all radiomics features and clinical and conventional MRI factors). The fitting formulas that were constructed with the training set were applied to the patients in the validation set.

### Statistical Analysis

Statistical analyses were performed with IBM SPSS Statistics (version 21.0). The consistency between observers for estimating ODs of infarct lesions, the DWI-ASPECTS score, Fazekas score, admission NIHSS, and the reliability of extracted radiomics features were evaluated by the inter-class correlation coefficient. An ICC value more than 0.75 was considered as good consistency.

A logistic regression was used (1) to compare the difference in each potential variable between the training and validation sets and (2) to select the significant demographic, clinical, and conventional MRI variables that associated with unfavorable outcome. A *P*-value less than 0.05 was considered statistically significant.

A receiver operating characteristic (ROC) curve was employed to assess the performance of the six prediction models for discriminating unfavorable outcome from favorable outcome both in the training and validation sets. The performance of a model was considered good when the value of area under the ROC curve (AUC) was larger than 0.75.

## Results

### Patients’ Characteristics

Due to imbalance of the data, we balanced them in both training and validation sets with Synthetic Minority Oversampling Technique (SMOTE) at first.

The basic characteristics of training and validation AIS patients are shown and compared in Table 1. There was no significant difference of outcome distribution between the two sets. The incidences of unfavorable outcome (mRS > 2 at 90 days) in the training and validation sets were 27.7% and 26.25% separately (*P* = 0.875). A univariate analysis showed that the following variables were significantly associated with the unfavorable outcome: age (*P* < 0.001), gender (*P* = 0.003), ODs (*P* = 0.006), admission NIHSS (*P* < 0.001), and DWI-ASPECTS (*P* < 0.001). Whereas, the time interval from stroke attack to MR examination (*P* = 0.119), hypertension (*P* = 0.748), diabetes mellitus (*P* = 0.276), hyperlipidemia (*P* = 0.567), coronary heart disease (*P* = 0.889), smoking (*P* = 0.646), alcohol consumption (*P* = 0.149), previous ictus (*P* = 0.877), and admission Fazekas score (*P* = 0.305) were not related to unfavorable outcome (Table 2). The average Fazekas score of the patients in the present study was 2.32. A multivariate analysis showed that the following variables were significantly associated with the unfavorable outcome: age (*P* < 0.001), gender (*P* = 0.010), admission NIHSS (*P* < 0.001) (Table 3).

**TABLE 3:** Logistic analyses for the predictors of unfavorable outcome of AIS patients.

**Characteristics**	**Univariate analysis**		**Multivariate analysis**	
	**OR (95% CI)**	***p*-value**	**OR (95% CI)**	***p*-value**
Age (>60.5 years)	1.067 (1.035, 1.099)	<0.001	1.070 (1.034, 1.107)	<0.001
Sex (male)	0.348 (0.175, 0.692)	0.003	0.336 (0.147, 0.770)	0.010
Admission NIHSS	1.244 (1.142, 1.356)	<0.001	1.270 (1.156, 1.394)	<0.001
Admission DWI-ASPECTS	0.627 (0.493, 0.789)	<0.001		
OD values of the lesion	1.037 (1.011, 1.065)	0.006		

*CI, confidence interval; DWI-ASPECTS, diffusion-weighted imaging-Alberta Stroke Program Early CT Score; NIHSS, National Institute of Health Stroke Scale; OD, orthogonal diameter; OR, odds ratio.*

### Assessment of Radiomics Features

**FIGURE 3:**
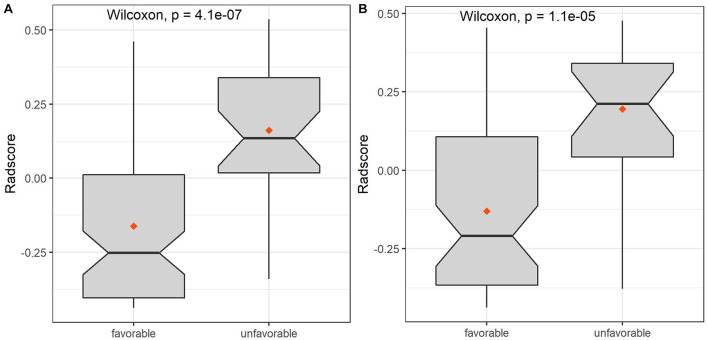
The box diagrams of radscore for discriminating favorable and unfavorable outcomes with combined model in the training and validation sets. **(A)** Training set. **(B)** Validation set.

**FIGURE 4:**
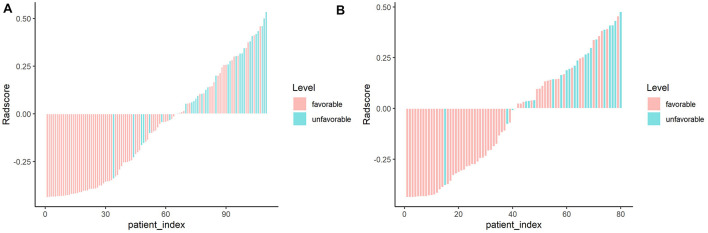
Waterfall chart showing radscores for each patient in the training and validation sets. The red columns represent patients with favorable outcome, whereas the green columns represent those with unfavorable outcome. **(A)** Training set. **(B)** Validation set.

The extracted features included three categories: shape features; first-order statistic features (histogram); and second-order statistic features, including gray level co-occurrence matrix (GLCM), gray level run-length matrix (GLRLM), gray level size zone matrix (GLSZM), gray level-dependent matrix (GLDM), and neighborhood gray-tone difference matrix (NGTDM). A total of 753 radiomics features were extracted from FLAIR and ADC images. Dimension reduction of data redundancy was performed with the Wilcoxon rank sum test, Spearman correlation analysis, and least absolute shrinkage and selection operator (LASSO). In brief, we employed cross-validation to determine an optimized tuning parameter λ when coefficients of indistinctive covariates were non-zero results. Then, this optimized λ was used for feature selection in the LASSO method (Li et al., 2018). At last, we used the selected features to setup a LASSO Cox regression model. After cutting off irrelevant and redundant features, the final six features were used as ultimate radiomics signatures: (1) DWI_wavelet. LH_first order_Interquartile Range; (2) DWI_wavelet. HL_GLCM_Idmn; (3) DWI_wavelet. HL_GLRLM_LongRun Emphasis; (4) FLAIR_original_GLSZM_ SmallArea Low GrayLevel Emphasis; (5) FLAIR_log. sigma. 5.0.mm. 3D_GLRLM_RunLength NonUniformity; and (6) FLAIR_wavelet. LL_GLDM_Low GrayLevel Emphasis. The detailed explanation of these features is shown in Supplementary Material 1. There was a significant difference of radscore between favorable and unfavorable outcome patients both in training and validation sets (Figures 3, 4 and Tables 4, 5).

**TABLE 4:** The diagnostic performance of the prediction models.

	**Models**	**AUC**	**95% CI**	**Sensitivity**	**Specificity**	**Accuracy**	**PPV**	**NPV**
Training (*n* = 110)	ADC	0.772	(0.681–0.864)	0.833	0.662	0.709	0.481	0.914
	FLAIR	0.731	(0.629, 0.834)	0.700	0.637	0.655	0.420	0.850
	ADC + FLAIR	0.815	(0.734, 0.895)	0.767	0.725	0.736	0.511	0.892
	Clin	0.791	(0.687, 0.894)	0.733	0.787	0.773	0.564	0.887
	Clin + Con MR	0.782	(0.684, 0.881)	0.800	0.637	0.682	0.453	0.895
	Combined all	0.926	(0.878, 0.974)	0.867	0.875	0.873	0.722	0.946
Validation (*n* = 80)	ADC	0.795	(0.687, 0.903)	0.810	0.525	0.600	0.378	0.886
	FLAIR	0.707	(0.583, 0.831)	0.857	0.576	0.650	0.419	0.919
	ADC + FLAIR	0.825	(0.726, 0.923)	0.857	0.644	0.700	0.462	0.927
	Clin	0.763	(0.632, 0.894)	0.667	0.763	0.738	0.500	0.896
	Clin + Con MR	0.751	(0.619, 0.883)	0.762	0.729	0.738	0.500	0.896
	Combined all	0.864	(0.773, 0.954)	0.810	0.746	0.762	0.531	0.917

*Combined all indicates all factors, including clinical and conventional MRI factors and radiomics features. AUC, area under the curves; ADC, apparent diffusion coefficient; Clin, clinical variables; Con MRI, conventional MRI factors; CI, confidence interval; FLAIR, fluid-attenuated inversion recovery; PPV, positive predictive value; NPV, negative predictive value.*

**TABLE 5:** Comparison the AUCs among different prediction models (*P*-value).

**Training set**					
**Training sets**	**FLAIR**	**ADC + FLAIR**	**Clin**	**Clin + Con MRI**	**All (combined model)**
ADC	0.458	0.190	0.795	0.877	<0.001*
FLAIR	–	0.041*	0.477	0.523	<0.001*
ADC + FLAIR	–	–	0.732	0.621	0.001*
Clin	–	–	–	0.555	0.003*
Clin + Con MRI	–	–	–	–	0.001*

**Validation Set**					
**Validation sets**	**FLAIR**	**ADC + FLAIR**	**Clin**	**Clin + Con MRI**	**All (combined model)**

ADC	0.210	0.609	0.693	0.586	0.221
FLAIR	–	0.036*	0.532	0.616	0.011*
ADC + FLAIR	–	–	0.471	0.393	0.446
Clin	–	–	–	0.323	0.031*
Clin + Con MRI	–	–	–	–	0.021*

All indicates all factors, including clinical and conventional MRI factors and radiomics features. AUC, area under the curves; ADC, apparent diffusion coefficient; Clin, clinical variables; Con MRI, conventional MRI factors; FLAIR, fluid-attenuated inversion recovery. *P < 0.05.

### Comparison of Performance of the Prediction Models

**FIGURE 5:**
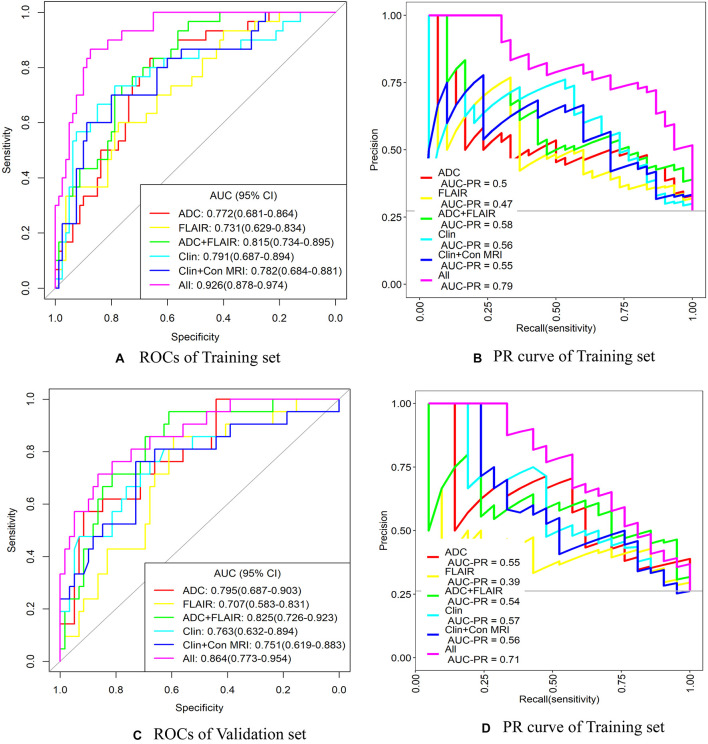
ROC and precision–recall (PR) curve analysis of the six models for predicting unfavorable outcome in training and validation sets. ADC, apparent diffusion coefficient; AUC, area under the curve; Clin + Con MRI, clinical + conventional MRI variables; CI, confidence interval; FLAIR, fluid-attenuated inversion recovery; ROC, receiver operating characteristic curve. **(A,B)** ROC and PR curves of the training set. **(C,D)** ROC and PR curves of the validation set.

The AUC values and other diagnostic performance indexes, including specificity, sensitivity, accuracy, positive and negative predictive values (PPV and NPV), and the comparison of AUCs among these models for predicting unfavorable outcome in the training and testing sets, are shown in Figure 5 and Tables 4, 5.

In the training set for predicting unfavorable outcome, the AUCs were as follows: ADC radiomics model 0.772, FLAIR radiomics model 0.731, ADC + FLAIR radiomics model 0.815, clinical model 0.791, and clinical and conventional MRI model 0.782. When combining clinical and conventional MRI factors and radiomics signatures, the AUC was significantly increased, reaching to 0.926 (Table 4 and Figure 5). In comparison, the combined model (all factors) had significantly better performance than any other model (*P* < 0.05) in the training set, so did the ADC + FLAIR model comparing with the FLAIR model (*P* = 0.041). However, there was no significant difference between the AUCs of other models (Table 5).

In the validation set for predicting unfavorable outcome, the AUCs were as follows: ADC radiomics model 0.795, FLAIR radiomics model 0.707, ADC + FLAIR radiomics model 0.825, clinical model 0.763, and clinical and conventional MRI model 0.751. When combining clinical and conventional MRI factors and radiomics signatures, the AUC value was increased to 0.864 (Table 4 and Figure 5). The PPV was relatively lower (0.531) and the NPV was relatively higher (0.917) in the validation set. In comparison, the combined model (all factors) had significantly better performance than FLAIR, clinical (Clin), and Clin + conventional (Con) MRI models (*P* < 0.05) in the validation set, so did the ADC + FLAIR model comparing with the FLAIR model (*P* = 0.036). However, there was no significant difference between the AUCs of other models (Table 5).

The diagnostic performance of the prediction models was tested with a precision–recall (PR) curve. The areas under curve of the “all” model (combined model) were largest both for ROCs and PR curves in training and validation sets (the areas under curve of PR was 0.79 in the training set and 0.71 in the validation set) (Figure 5). This indicated that the combined model could attain better diagnostic performance than any other models.

In the combined model (“all” model), the ORs for various factors were as follows: radioscore (ADC + FLAIR), 2.08; age (>60.5 years), 1.07; sex (male), 0.22; DWI-ASPECTS ≥ 7, 1.25; and admission NIHSS, 1.18 (Supplementary Material 2). Thus, radioscore, age, DWI-ASPECTS ≥ 7, and admission NIHSS were the risk factors of unfavorable outcome in AIS patients.

## Discussion

In this study, we extracted radiomics features based on FLAIR and ADC images and developed a combination model for predicting the functional outcome in AIS patients. We found that the radiomics signatures, especially those extracted from ADC image, were associated with unfavorable outcome (mRS > 2) and was a value risk factor. Moreover, radiomics based on FLAIR and ADC can improve the diagnostic performance of the combination prediction model.

Conventional MRI, including FLAIR and DWI, had been proven a useful tool in predicting AIS outcome. The location, volume, as well as signal intensity of infarct lesions are the effective markers of prognosis. Boss et al. (2019) explored the influence of visual DWI lesion homogeneity on clinical outcome in 30 AIS patients. A significant difference of mRS scores was found between patients with homogenous and non-homogeneous DWI lesions. However, they did not offer a quantization parameter of lesion homogeneity, and the finding has not been verified in other studies. In another study that included 65 AIS patients treated with thrombolysis, Tanriverdi et al. (2016) assessed the relationship between FLAIR hyperintensity and functional outcome. They suggested that a rise of FLAIR intensity ratio is a marker of favorable outcome. Whereas, the setup of prediction model and validation in other medical centers had not been made. Radiomics capture subtle variation within medical images. After extraction of radiomics features and dimension reduction of data redundancy, the strongest features could be used to analyze the heterogeneity of lesions and thus to predict patient outcome (Qiu et al., 2019; Tomaszewski and Gillies, 2021). Radiomics is superior to conventional imaging visual analysis in identifying the heterogeneity of AIS lesions (Wen et al., 2020). Qiu et al. (2019) also found that the radiomics features extracted from non-contrast CT and CT angiography were useful to predict the recanalization of cerebral arteries with intravenous injection of alteplase in AIS patients. Therefore, we selected FLAIR and DWI images to develop radiomics signatures and explore their prognostic role of clinical outcome in AIS patients.

Our results suggested that radiomics signatures extracted from FLAIR and DWI can be correlated with mRS scores. The diagnostic odds ratio (OR) analysis also showed that the weight of radioscore (2.08) was higher than any other clinical and conventional MRI factors (from 0.22 to 1.25). This result indicated that radioscore is the most important risk factor for predicting unfavorable outcome of AIS patients. It confirms our hypothesis that these features could be used as a biomarker to predict AIS outcome. In the study conducted by Tozer et al. (2018), texture features were extracted from FLAIR images. They found that these texture features were correlated with executive dysfunction and cognition in patients with cerebral small vessel disease instead of AIS. Wang et al. (2020) also confirmed that texture features extracted from FLAIR images and ADC maps could serve as a biomarker of stroke severity. While these features were not predictive of mRS scores in their study, on the other hand, they only enrolled ischemic stroke patients in subacute phase and did not test their results in an external validation set. In contrast, in the present study, we enrolled the AIS patients from three hospitals who suffered from ischemic attack less than 72 h and extracted radiomics signatures based both on FLAIR and ADC images.

Although radiomics features capture subtle heterogeneity beyond the perception of the human eye, our study showed that the AUCs of ADC + FLAIR radiomics feature model were slightly superior to those of pure Clin or Clin + Con MRI model both in the training and validation sets. But, other diagnostic performance indexes of ADC + FLAIR radiomics feature model were similar or even slightly lower than those of pure Clin or Clin + Con MRI model both in the training and validation sets. Thus, the direct usefulness of models only based on ADC and FLAIR radiomics features to predict unfavorable outcome of AIS may cause bias or errors. On the other hand, the multi-parameter analysis has been proven to attain better diagnostic performance. Therefore, the model integrating clinical and conventional MRI factors could be used in predicting outcome in AIS patients. The ability for predicting AIS outcome was improved significantly for this combination. In our study, the AUC and PPV of predicting unfavorable outcome in the training set with combined model were improved significantly (0.926 and 0.722, separately). Previous studies have also reported diagnostic performance improvement when this methodology was combined to clinical and conventional imaging variables. In one retrospective study of 38 pathologically confirmed intraductal papillary mucinous neoplasms (IPMN) of the pancreas, Permuth et al. (2016) found that the combined radiogenomic model attained more accurate prediction of the IPMN malignancy (AUC = 0.92). Wen et al. (2020) extracted radiomics features from non-contrast CT and CTA images of 126 patients with MCA infarct. They developed a combined prediction model based on radiomics and ASPECTS and showed a good performance of this model in predicting malignant MCA infarct both in the training and validation sets (with AUC of 0.917 and 0.913, respectively). These results confirmed the superiority of a combined model, indicating that clinical and conventional imaging factors and radiomics features have an intercrossing incremental effect on each other, adding up to a more satisfactory outcome prediction model for AIS patients. Thus, radiomics features should be combined to multi-parameter analysis with clinical variables as well as conventional visual factors to form a comprehensive marker panel with better performance. However, the PPV was relatively lower (0.531) and the NPV was relatively higher (0.917) in the validation set. This means four participants in the validation set will be incorrectly labeled as unfavorable outcome. The phenomena of relatively lower PPV and higher NPV in the validation set of the present study is probably because the data of the validation set come from the other two institutes. The heterogeneity of MR data and clinical factors could reduce the efficiency of the combined model.

Clinical and MR factors also play an important role in outcome prediction of AIS. Previous studies have reported that the clinical variables, such as gender, age, and admission NIHSS, could be used as factors for predicting the outcome of AIS patients (Kim and Vemuganti, 2015; Wu et al., 2019). To validate these results, we used the 90-day mRS > 2 as an unfavorable outcome criteria. In our study, multivariate logistic analysis confirmed that higher admission NIHSS scores, age (>60.5 years old), and gender (female) were independent predictors of unfavorable outcome. Both the above studies and the present study proved that the selected clinical variables have the potential in predicting long-term outcome of AIS. Along with clinical predictors, our findings also indicated that conventional MRI variables, including infarct volume and DWI-ASPECTS score, seem to be independent markers for predicting functional outcome. In previous studies, certain conventional MRI factors, especially those based on FLAIR and DWI images, have been proven to be associated with the outcome of AIS patients (Bucker et al., 2017; Liu et al., 2018; Tozer et al., 2018). Similarly, our earlier study also proved that with a cut-off value of 57.3 ml, the infarct volume could predict unfavorable patient outcome (Yuan et al., 2019). In the present study, we did not find an association between Fazekas score and 90-day mRS. This phenomena could ascribed to the lower Fazekas score of the patients in this study. The patients with lower Fazekas score experienced a less severe cerebral vascular disease (Liu et al., 2018). Otherwise, only moderate to severe leukoaraiosis (with higher Fazekas score) was the independent predictor of unfavorable outcome. Therefore, the use of clinical and conventional MR factors alone for predicting the outcome accurately has been challenged (Boss et al., 2019).

### Limitations

It should be noted that there are several limitations in the present study. The main limitation is the relatively small sample size, which might cause an overfitting problem for developing radscore. However, to address this limitation, we used the external validation analysis to minimize this bias. Second, we collected the imaging data of AIS retrospectively. This might lead to selection bias. The results obtained from the consecutive AIS patients enabled the development of tentative outcome prediction model. Third, our current study did not include functional MR imaging. In the future, the data of perfusion imaging, amide proton transfer imaging, and other functional imaging could be added to a comprehensive model. Finally, only patients who had a stroke in the MCA territory are included, and this analysis cannot be generalized to strokes in other areas of the brain. However, infarction in the MCA territory is a most common ischemic stroke with high prevalence of sequelae and mortality (Yuan et al., 2019; Wen et al., 2020). Thus, the prediction model combining radiomics features and clinical and conventional MRI factors may still facilitate the early and accurate prediction of stroke. We deduce that the radiomics features extracted from ADC and FLAIR can be helpful in the development of clinical decisions of AIS patients, especially for those patients with stroke in the MCA territory.

## Conclusion

Our results showed the usefulness of radiomics based on FLAIR and ADC in predicting unfavorable functional outcome in patients with AIS. Radiomics can be added as an independent predictor along with clinical and conventional MRI factors. The results proved that the combination model, which incorporates clinical variables, conventional MRI information, and radiomics, demonstrated the highest efficiency in the prediction of functional outcomes after AIS attack. This model can facilitate prediction of AIS evolution in acute phase and would contribute more to clinical evaluation process.

## Data Availability Statement

The raw data supporting the conclusions of this article will be made available by the authors, without undue reservation.

## Ethics Statement

The studies involving human participants were reviewed and approved by The Ethics Committees of The Second Hospital of Hebei Medical University. The ethics committee waived the requirement of written informed consent for participation.

## Author Contributions

GQ, RB, J-LR, WW, SD, and TY made a substantial contribution to the concept and design, acquisition of data or analysis, and interpretation of data. GQ, YL, and TY drafted the manuscript and revised it critically for relevant intellectual content. GQ, RB, and SD performed the MR examination and follow-up of patients. All the authors approved the final version of the manuscript.

## Conflict of Interest

J-LR was employed by GE Healthcare China. The remaining authors declare that the research was conducted in the absence of any commercial or financial relationships that could be construed as a potential conflict of interest.

## Publisher’s Note

All claims expressed in this article are solely those of the authors and do not necessarily represent those of their affiliated organizations, or those of the publisher, the editors and the reviewers. Any product that may be evaluated in this article, or claim that may be made by its manufacturer, is not guaranteed or endorsed by the publisher.

## Footnotes

1
http://www.itk-snap.org

2
http://download.slicer.org

3
http://www.radiomicss.io

## References

[B1] Boss S. M. Moustafa R. R. Moustafa M. A. Sadek A. E. Mostafa M. M. Aref H. M. (2019). Lesion homogeneity on diffusion-weighted imaging is a marker of outcome in acute ischemic stroke. *Egyptian J. Neurol. Psychiatry Neurosurg.* 55:59. 10.1186/s41983-019-0101-z

[B2] Bucker A. Boers A. M. Bot J. C. J. Berkhemer O. A. Lingsma H. F. Yoo A. J. (2017). Associations of ischemic lesion volume with functional outcome in patients with acute ischemic stroke : 24-hour versus 1-week imaging. *Stroke* 48 1233–1240. 10.1161/STROKEAHA.116.015156 28351963

[B3] Choi V. Kate M. Kosior J. C. Buck B. Steve T. McCourt R. (2015). National Institutes of Health Stroke Scale score is an unreliable predictor of perfusion deficits in acute stroke. *Int. J. Stroke* 10 582–588. 10.1111/ijs.12438 25845906

[B4] Collins G. S. Reitsma J. B. Altman D. G. Moons K. G. M. (2015). Transparent Reporting of a multivariable prediction model for Individual Prognosis Or Diagnosis (TRIPOD): the TRIPOD Statement. *Br. J. Surg.* 102 148–158. 10.1016/j.eururo.2014.11.025 25627261

[B5] Darwish E. A. Abdelhameed-El-Nouby M. Geneidy E. (2020). Mapping the ischemic penumbra and predicting stroke progression in acute ischemic stroke: the overlooked role of susceptibility weighted imaging. *Insights Imaging* 11 6–17. 10.1186/s13244-019-0810-y 31930428 PMC6955386

[B6] Dolotova D. Arkhipov I. Blagosklonova E. Donitova V. Barmina T. Sharifullin F. (2020). Application of radiomics in vesselness analysis of CT angiography images of stroke patients. *Stud. Health Technol. Inform.* 270 33–37. 10.3233/SHTI200117 32570341

[B7] Fazekas F. Barkhof F. Wahlund L. O. Pantoni L. Erkinjuntti T. Scheltens P. (2002). CT and MRI rating of white matter lesions. *Cerebrovasc. Dis.* 13 31–36. 10.1159/000049147 11901240

[B8] Ichijo M. Miki K. Ishibashi S. Tomita M. Kamata T. Fujigasaki H. (2013). Posterior cerebral artery laterality on magnetic resonance angiography predicts long-term functional outcome in middle cerebral artery occlusion. *Stroke* 44 512–515. 10.1161/STROKEAHA.112.674101 23192760

[B9] Jiang L. Chen Y. C. Zhang H. Peng M. Y. Chen H. Y. (2019). FLAIR vascular hyperintensity in acute stroke is associated with collateralization and functional outcome. *Eur. Radiol.* 29 4879–4888. 10.1007/s00330-019-06022-0 30762112

[B10] Kassner A. Liu F. Thornhill R. E. Tomlinson G. Mikulis D. J. (2009). Prediction of hemorrhagic transformation in acute ischemic stroke using texture analysis of postcontrast T1-weighted MR images. *J. MagnReson. Imaging* 30 933–941. 10.1002/jmri.21940 19856407

[B11] Kim T. H. Vemuganti R. (2015). Effect of sex and age interactions on functional outcome after stroke. *CNS Neurosci. Ther.* 21 327–336. 10.1111/cns.12346 25404174 PMC6495347

[B12] Li W. Zhang L. Tian C. Song H. Fang M. Hu C. (2018). Prognostic value of computed tomography radiomics features in patients with gastric cancer following curative resection. *Eur. Radiol.* 29 3079–3089. 10.1007/s00330-018-5861-9 30519931

[B13] Liu Y. Y. Zhang M. Chen Y. Gao T. Yun W. W. Zhou X. J. (2018). The degree of leukoaraiosis predicts clinical outcomes and prognosis in patients with middle cerebral artery occlusion after intravenous thrombolysis. *Brain Res.* 1681 28–33. 10.1016/j.brainres.2017.12.033 29288062

[B14] Permuth J. B. Choi J. Balarunathan Y. Kim J. Chen D. T. Chen L. (2016). Combining radiomic features with a miRNA classifier may improve prediction of malignant pathology for pancreatic intraductal papillary mucinous neoplasms. *Oncotarget* 7 85785–85797. 10.18632/oncotarget.11768 27589689 PMC5349874

[B15] Qiu W. Kuang H. Nair J. Assis Z. Najm M. McDougall C. (2019). Radiomics-based intracranial thrombus features on CT and CTA predict recanalization with intravenous alteplase in patients with acute ischemic stroke. *AJNR Am. J. Neuroradiol.* 40 39–44. 10.3174/ajnr.A5918 30573458 PMC7048606

[B16] Rudilosso S. Olivera M. Esteller D. Laredo C. Amaro S. Llull L. (2021). Susceptibility vessel sign in deep perforating arteries in patients with recent small subcortical infarcts. *J. Stroke Cerebrovasc. Dis.* 30:105415. 10.1016/j.jstrokecerebrovasdis.2020.105415 33142246

[B17] Sommer P. Posekany A. Serles W. Marko M. Scharer S. Fertl E. (2018). Is functional outcome different in posterior and anterior circulation stroke? *Stroke* 49 2728–2732. 10.1161/STROKEAHA.118.021785 30355215

[B18] Tang T. T. Jiao Y. Cui Y. Zhao D. L. Zhang Y. Wang Z. (2020). Penumbra-based radiomics signature as prognostic biomarkers for thrombolysis of acute ischemic stroke patients: a multicenter cohort study. *J. Neurol.* 267 1454–1463. 10.1007/s00415-020-09713-7 32008072

[B19] Tanriverdi Z. Gocmen R. Oguz K. K. Topcuoglu M. A. Arsava E. M. (2016). Elevations in tissue fluid-attenuated inversion recovery signal are related to good functional outcome after thrombolytic treatment. *J. Stroke Cerebrovasc. Dis.* 25 480–483. 10.1016/j.jstrokecerebrovasdis.2015.10.024 26652209

[B20] Tei H. Uchiyama S. Usui T. Ohara K. (2011). Diffusion-weighted ASPECTS as an independent marker for predicting functional outcome. *J. Neurol.* 258 559–565. 10.1007/s00415-010-5787-x 20957383

[B21] Tomaszewski M. R. Gillies R. J. (2021). The biological meaning of radiomic features. *Radiology* 2021:202553. 10.1148/radiol.2021202553 33900879 PMC8906340

[B22] Tozer D. J. Zeestraten E. Lawrence A. J. Barrick T. R. Markus H. S. (2018). Texture analysis of T1-weighted and fluid-attenuated inversion recovery images detects abnormalities that correlate with cognitive decline in small vessel disease. *Stroke* 49 1656–1661. 29866751 10.1161/STROKEAHA.117.019970 PMC6022812

[B23] van Griethuysen J. J. M. Fedorov A. Parmar C. Hosny A. Aucoin N. Narayan V. (2017). Computational radiomics system to decode the radiographic phenotype. *Cancer Res.* 77 e104–e107. 10.1158/0008-5472.CAN-17-0339 29092951 PMC5672828

[B24] Wang H. Lin J. Zheng L. Zhao J. Song B. Dai Y. M. (2020). Texture analysis based on ADC maps and T2-FLAIR images for the assessment of the severity and prognosis of ischaemic stroke. *Clin. Imaging* 67 152–159. 10.1016/j.clinimag.2020.06.013 32739735

[B25] Wen X. Li Y. He X. Xu Y. Shu Z. Hu X. (2020). Prediction of malignant acute middle cerebral artery infarct via computed tomography radiomics. *Front. Neurosci.* 14:708. 10.3389/fnins.2020.00708 32733197 PMC7358521

[B26] Wu X. Liu G. Zhou W. Ou A. H. Liu X. Wang Y. H. (2019). Outcome prediction for patients with anterior circulation acute ischemic stroke following endovascular treatment: a single-center study. *Exp. Ther. Med.* 18 3869–3876. 10.3892/etm.2019.8054 31641377 PMC6796376

[B27] Yuan T. Ren G. Quan G. Liu Y. (2019). Maximum lesions area and orthogonal values accessed from DWI images would be alternative imaging markers for predicting the outcome of acute ischemia in the middle cerebral artery territory. *Acta Radiol.* 60 628–633. 10.1177/0284185118795330 30130971

