# Supplementary figures and images for: FLAIR and ADC Image-Based Radiomics Features as Predictive Biomarkers of Unfavorable Outcome in Patients With Acute Ischemic Stroke
Source: Front Neurosci. 2021 Sep 16;15:730879. doi: 10.3389/fnins.2021.730879 (PMC8483716; doi:10.3389/fnins.2021.730879)

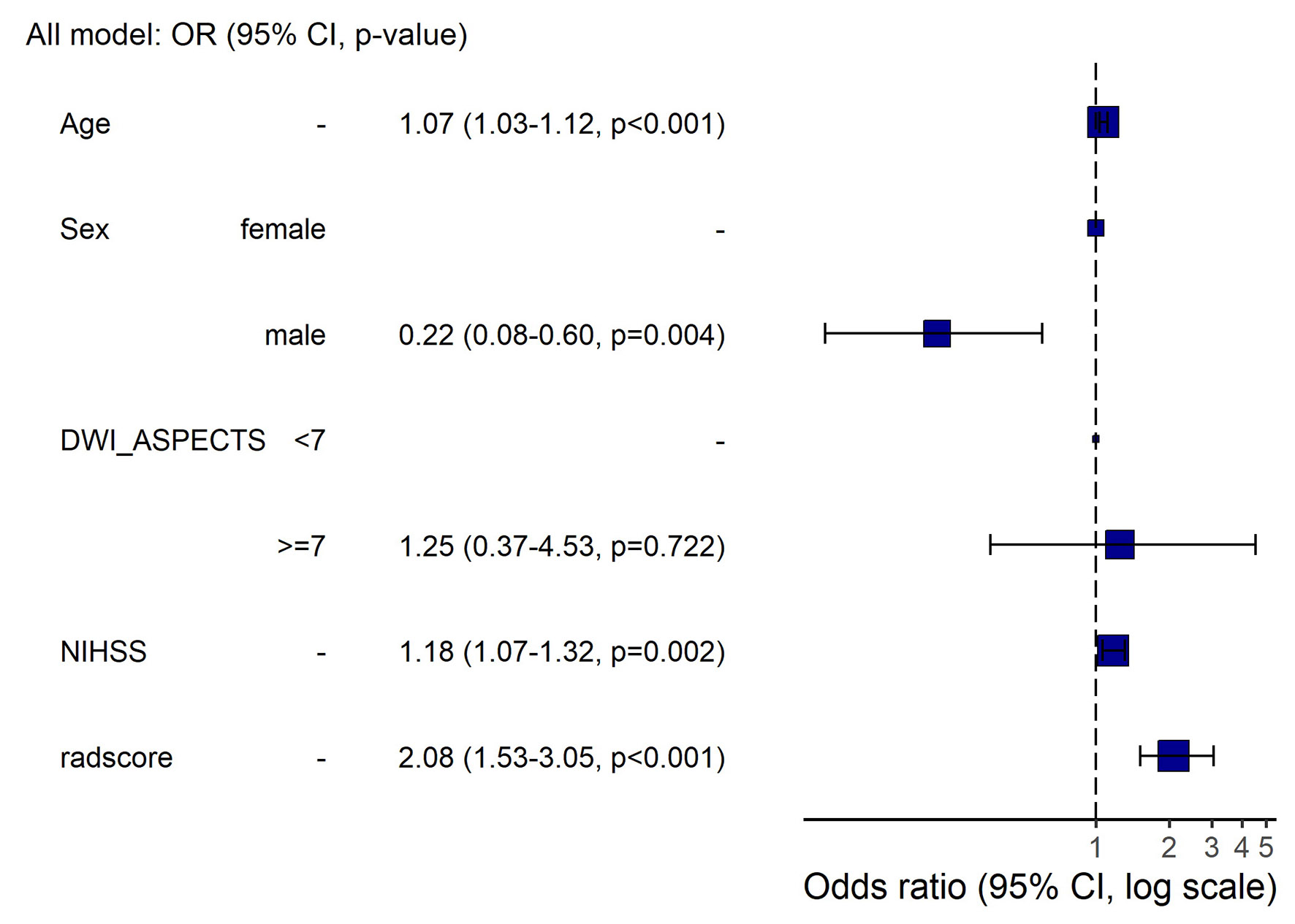

Supplement: Supplementary file 1 [file Image_1.JPEG]
