# Supplementary material for: FLAIR and ADC Image-Based Radiomics Features as Predictive Biomarkers of Unfavorable Outcome in Patients With Acute Ischemic Stroke
Source: Front Neurosci. 2021 Sep 16;15:730879. doi: 10.3389/fnins.2021.730879 (PMC8483716; doi:10.3389/fnins.2021.730879)
Supplement: Supplementary file 2 [file Table_1.DOCX]

**Supplement TABLE 1 ⎜ The characteristic of 6 final features for Radiomics assessment.**

| **Image type** | **Filter type** | **Filter parameter** | **Feature classes** | **Feature name** | **Equation** | **Notes** |
| --- | --- | --- | --- | --- | --- | --- |
| **ADC** | **Wavelet** | **LH** | **First order** | **Interquartile Range** | $\mathbf{P}_{\mathbf{75}}\mathbf{-}\mathbf{P}_{\mathbf{25}}$ | $\mathbf{P}_{\mathbf{25}}\mathbf{and}\mathbf{P}_{\mathbf{75}}$ **are the 25th and 75th percentile of the image array** |
| **ADC** | **Wavelet** | **HL** | **GLCM** | **Inverse Difference Moment Normalized (IDMN)** | $\sum_{\mathbf{k=0}}^{\mathbf{N}_{\mathbf{g}}\mathbf{-1}} \frac{\mathbf{p}_{\mathbf{x-y}}\left( \mathbf{k} \right)}{\mathbf{1+}\left( \frac{\mathbf{k}^{\mathbf{2}}}{\mathbf{N}_{\mathbf{g}}^{\mathbf{2}}} \right)}$ | $\mathbf{N}_{\mathbf{g}}$ **be the number of discrete intensity levels in the image.** $\mathbf{p}_{\mathbf{x-y}}\left( \mathbf{k} \right)\mathbf{=}\sum_{\mathbf{i=1}}^{\mathbf{N}_{\mathbf{g}}} \sum_{\mathbf{j=1}}^{\mathbf{N}_{\mathbf{g}}} \mathbf{p}\left( \mathbf{i,j} \right)$ |
| **ADC** | **Wavelet** | **HL** | **GLRLM** | **Long Run Emphasis (LRE)** | $\frac{\sum_{\mathbf{i=1}}^{\mathbf{N}_{\mathbf{g}}} \sum_{\mathbf{j=1}}^{\mathbf{N}_{\mathbf{r}}} \mathbf{P}\left( \mathbf{i,j} \vert\boldsymbol{\theta} \right)\mathbf{j}^{\mathbf{2}}}{\mathbf{N}_{\mathbf{r}}\left( \boldsymbol{\theta} \right)}$ | $\mathbf{N}_{\mathbf{r}}$ **be the number of discreet run lengths in the image.** $\mathbf{N}_{\mathbf{r}}\left( \boldsymbol{\theta} \right)$ **be the number of runs in the image along angle** $\boldsymbol{\theta}$ |
| **FLAIR** | **LoG** | **5.0mm** | **GLRLM** | **Run Length Non-Uniformity (RLN)** | $\frac{\sum_{\mathbf{j=1}}^{\mathbf{N}_{\mathbf{r}}} \left( \sum_{\mathbf{i=1}}^{\mathbf{N}_{\mathbf{g}}} \mathbf{P}\left( \mathbf{i,j} \vert\boldsymbol{\theta} \right) \right)^{\mathbf{2}}}{\mathbf{N}_{\mathbf{r}}\left( \boldsymbol{\theta} \right)}$ |  |
| **FLAIR** | **Wavelet** | **LL** | **GLDM** | **Low Gray Level Emphasis (LGLE)** | $\frac{\sum_{\mathbf{i=1}}^{\mathbf{N}_{\mathbf{h}}} \sum_{\mathbf{j=1}}^{\mathbf{N}_{\mathbf{d}}} \frac{\mathbf{P}\left( \mathbf{i,j} \right)}{\mathbf{i}^{\mathbf{2}}}}{\mathbf{N}_{\mathbf{z}}}$ | $\mathbf{N}_{\mathbf{z}}$ **be the number of dependency zones in the image** |
| **FLAIR** | **-** | **-** | **GLSZM** | **Small Area Low Gray Level Emphasis (SALGLE)** | $\frac{\sum_{\mathbf{i=1}}^{\mathbf{N}_{\mathbf{g}}} \sum_{\mathbf{j=1}}^{\mathbf{N}_{\mathbf{s}}} \frac{\mathbf{P}\left( \mathbf{i.j} \right)\mathbf{i}^{\mathbf{2}}}{\mathbf{i}^{\mathbf{2}}\mathbf{j}^{\mathbf{2}}}}{\mathbf{N}_{\mathbf{z}}}$ | $\mathbf{N}_{\mathbf{s}}$ **be the number of discreet zone sizes in the image** |

***GLCM: Gray Level Cooccurence Matrix; GLDM: Gray Level Dependence Matrix; GLRLM: Gray Level Run Length Matrix; GLSZM: Gray Level Size Zone Matrix; LoG: Laplace of Gaussian.***
