# Supplementary material for: FLAIR and ADC Image-Based Radiomics Features as Predictive Biomarkers of Unfavorable Outcome in Patients With Acute Ischemic Stroke
Source: Front Neurosci. 2021 Sep 16;15:730879. doi: 10.3389/fnins.2021.730879 (PMC8483716; doi:10.3389/fnins.2021.730879)
Supplement: Supplementary file 3 [file Table_2.DOCX]

**Supplement TABLE 2 ⎜ Comparison the AUCs among different prediction models （*P* value）**

| **Training Set** | | | | | |
| --- | --- | --- | --- | --- | --- |
| **Training sets** | **FLAIR** | **ADC+FLAIR** | **Clin** | **Clin+ Con MRI** | **All (combined model)** |
| ADC | 0.458 | 0.190 | 0.795 | 0.877 | <0.001* |
| FLAIR | _ | 0.041* | 0.477 | 0.523 | <0.001* |
| ADC+FLAIR | _ | _ | 0.732 | 0.621 | 0.001* |
| Clin | _ | _ | _ | 0.555 | 0.003* |
| Clin+ Con MRI | _ | _ | _ | _ | 0.001* |
| **Validation Set** | | | | | |
| **Validation sets** | **FLAIR** | **ADC+FLAIR** | **Clin** | **Clin+ Con MRI** | **All (combined model)** |
| ADC | 0.210 | 0.609 | 0.693 | 0.586 | 0.221 |
| FLAIR | _ | 0.036* | 0.532 | 0.616 | 0.011* |
| ADC+FLAIR | _ | _ | 0.471 | 0.393 | 0.446 |
| Clin | _ | _ | _ | 0.323 | 0.031* |
| Clin+ Con MRI | _ | _ | _ | _ | 0.021* |

*All, all factors, including clinical, conventional MRI factors and radiomics features; AUC, area under the curves; ADC,* *apparent diffusion coefficient; Clin，clinical variables；Con MRI, conventional MRI factors; FLAIR, fluid-attenuated inversion recovery.*
